# Extracellular domains of E-cadherin determine key mechanical phenotypes of an epithelium through cell- and non-cell-autonomous outside-in signaling

**DOI:** 10.1371/journal.pone.0260593

**Published:** 2021-12-22

**Authors:** Darwesh Mohideen Kaderbatcha Aladin, Yeh Shiu Chu, Shuo Shen, Robert Charles Robinson, Sylvie Dufour, Virgile Viasnoff, Nicolas Borghi, Jean Paul Thiery

**Affiliations:** 1 Mechanobiology Institute, National University of Singapore, Singapore, Singapore; 2 Institute of Molecular and Cell Biology, A*STAR, Singapore, Singapore; 3 BioSyM Interdisciplinary Research Group, Singapore-MIT Alliance for Research and Technology (SMART), Singapore, Singapore; 4 Sinopharm, Zhengdian, Jiangxia District, Wuhan, Hubei, China; 5 IMRB, Université Paris Est Créteil, INSERM, Créteil, France; 6 UMI 3639 CNRS, Singapore; 7 Institut Jacques Monod, Université de Paris, CNRS, Paris, France; 8 Guangzhou Laboratory, International Bioisland, Guangzhou, Haizhu District, China; University of Illinois at Chicago, UNITED STATES

## Abstract

Cadherins control intercellular adhesion in most metazoans. In vertebrates, intercellular adhesion differs considerably between cadherins of type-I and type-II, predominantly due to their different extracellular regions. Yet, intercellular adhesion critically depends on actomyosin contractility, in which the role of the cadherin extracellular region is unclear. Here, we dissect the roles of the Extracellular Cadherin (EC) Ig-like domains by expressing chimeric E-cadherin with E-cadherin and cadherin-7 Ig-like domains in cells naturally devoid of cadherins. Using cell-cell separation, cortical tension measurement, tissue stretching and migration assays, we show that distinct EC repeats in the extracellular region of cadherins differentially modulate epithelial sheet integrity, cell-cell separation forces, and cell cortical tension with the Cdc42 pathway, which further differentially regulate epithelial tensile strength, ductility, and ultimately collective migration. Interestingly, dissipative processes rather than static adhesion energy mostly dominate cell-cell separation forces. We provide a framework for the emergence of epithelial phenotypes from cell mechanical properties dependent on EC outside-in signaling.

## Introduction

Intercellular adhesion plays an important role in the development and maintenance of multicellular organisms [1–3]. Such adhesion is established and maintained through calcium-dependent, cell-cell adhesion molecules known as cadherins. Type-I and type-II cadherins, also known as classical cadherins, form one of six highly conserved branches of the cadherin superfamily, classified based on protein sequences in the extracellular cadherin (EC) domains. Classical cadherins typically have an extracellular region comprising five EC repeats (EC1-5), a single transmembrane region and a cytoplasmic region [4, 5]. They are often found concentrated in adherens junctions, dynamically interacting with the contractile cytoskeleton through catenin complexes. Despite striking structural similarities, E-cadherin (type-I) engenders robust intercellular adhesion, as seen in the epithelium, whereas cadherin-7 (type-II) is associated with much weaker adhesion, such as that in the mesenchyme [6, 7]. This phenotypic difference may be attributed to the cadherin extracellular region. Indeed, whereas all three regions of cadherins are essential for normal function, the EC region dictates differences in cell-cell separation forces (SFs)—a metric used as a proxy for intercellular adhesion energy [8]—between type-I and type-II cadherin-expressing cell pairs [6].

In E-cadherin (E-cad) EC domains, henceforth referred to as EECs, the first and outermost EC domain (EEC1) has a tryptophan residue at position 2 (Trp2), which interacts with a hydrophobic pocket in the opposing EEC1 of its cadherin pair to form strand-swap dimers. In contrast, in cadherin-7 (cad-7) EC domains, henceforth referred to as 7ECs, the conserved Trp2 and Trp4 residues in 7EC1 anchor into a larger hydrophobic pocket to form two strand-swaps [9]. Associations and dissociations of strand-swapped dimers were recently identified to be mediated by binding intermediates referred to as X-dimers, which form through extensive surface interactions between the residues near the EC1-2 calcium binding sites [10–12]. X-dimers behave as catch bonds [13], while strand-swap dimers form slip bonds. Structural and biophysical studies corroborate that cadherins tune their *trans*-bond lifetimes by switching between X- and strand-swap dimer states [14]. As both type-I and type-II cadherins are known to form X-dimers, whether the strand-swapping specifics of type-I and–II cadherins, or other causes are responsible for the significant difference between E-cad and cad-7 mediated cell-cell SFs remains to be uncovered [10, 14, 15].

Indeed, although the residues involved in strand-swap and X-dimerizations are exclusive to EC1,2 domains, mounting evidence suggests that the modular cadherin structure can also engage in multiple, *trans*- and *cis-*bonds involving more than the outer two EC Ig-like domains [16–24]. Moreover, *in silico* studies show that EEC and 7EC domains unfold in remarkably different ways under mechanical forces [25]. This raises the possibility that differences in adhesive properties between type-I and type-II cadherins arise from other ECs than EC1,2, and do not rely uniquely on strand-swap or X-dimerizations.

Dual pipette cell-cell SF assays revealed that, after a few minutes of initial cell-cell contact, cell-cell SF is crucially dependent on the actin cytoskeletal anchorage of cadherins [26], in a cadherin type-dependent manner, and this is further supported by observations that cadherins uncouple from the cytoskeleton rather than from each other in zebrafish embryo cell-cell separation [27]. Indeed, cadherins appear to contribute to intercellular adhesion energy by locally down-regulating actomyosin cortical contractility [28–30], with marginal contributions from the molecular binding energy of *trans*-interacting E-cad between cells [27, 31, 32]. Nevertheless, myosin-II–driven actin dynamics do modulate the immobilization of E-cad at the cell-cell contact periphery in a mechanosensitive manner [33], where cadherin *trans*-interactions are required to physically link cells together [27, 29]. Whether specific ECs of type-I and -II cadherins differentially regulate the cortical actomyosin cytoskeleton, and whether such a regulation depends on trans-interactions remain to be demonstrated.

In this work, we thus sought to address how the distinct ECs of type-I and -II cadherins regulate cell-cell adhesive properties, the contributions of trans-interactions and their effects on cortical mechanics, and the consequences at the tissue level. To do so, we examined the properties of cells individually expressing four different chimeric cadherins whose five EC regions are combinations of EC domains from E-cad or cad-7. Each chimera retained the E-cad transmembrane and cytoplasmic regions to preserve the interactions of E-cad, the catenin complex, and the cytoskeleton. We found that epithelial-like phenotype remained after exchanging EEC1-3 to 7EC1-3 but was fully lost upon exchange of the 4 first domains. Using a novel pillar-pipette assay to measure cell-cell SFs, we found that the exchange of the sole EEC3 by 7EC3 lead to a very strong decrease of SF, and the replacement of the 3 domains EEC1-3 drastically reduced SF maturation. Next, we determined by pipette aspiration that EEC4 and EEC1-3 antagonistically affected CdC42 activity and cell cortical tension cell-autonomously. From this, and the measurement of cell-cell compaction, we determined that the static adhesion energy was a minor contributor to cell-cell SF, which was therefore mostly governed by dissipative processes to which EC region stretching before trans-bond rupture quantitatively suffices to contribute. Finally, using a custom substrate-free tissue stretcher, we provide evidence that EEC3 offers tensile strength to cell sheets, while EEC1-3 provides ductility, in accordance with their respective effects on cell-cell SF and cortical tension. We propose that the antagonistic effects of tissue ductility and cohesion endowed by the different EC regions ultimately determine the speed of collective cell migration.

## Materials and methods

### Cell lines, constructs, lentivirus-based transductions, and immunostaining

Protein sequences and domain organizations of mouse E-cad and cadherin-7 were used to construct chimeric cadherins, including 77EEE, 777EE, 7777E and EE7EE. In these chimeras, we preserved the calcium binding sites. Sequences were cloned into PLVX-puro vector with eGFP at the C-terminus and proper insertion confirmed by sequencing. HEK293T cells were co-transfected (Effectene transfection reagent) with the plasmid and optimized lentiviral packaging mix (ViraPower^™^). The viral supernatant was harvested, filtered, and added to S180 cells plated at 80% confluence. Stably transduced cells were selected based on puromycin resistance, FACS sorted for a moderate level of eGFP expression, and checked for mycoplasma contamination (MycoAlert^™^, Lonza). Rabbit anti-α-catenin (Sigma-Aldrich), mouse monoclonal anti-β-catenin, mouse anti-p120-catenin (Invitrogen) and mouse monoclonal anti-vinculin (Sigma-Aldrich) were used for immunostaining. Strand-swap incompetent E-cad (W2A mutant) and X-dimer incompetent E-cad (K14E) were also stably expressed separately in S180 cells following the above lentivirus-based transduction protocol and FACS sorted for moderate expression.

### Computer model for validation of preserved domain interactions and calcium binding sites in chimeric cadherin ECs

Models of juxtaposed domains at the hybrid interfaces were built using the web-based interface of SWISS-MODEL [34] and existing protein structures as templates: 2A62, 3Q2V, 3Q2W, 1L3W and 5VEB [5, 9, 35, 36]. Calcium ions were placed by hand and sidechains angles were orientated to coordinate the ions in Coot [37].

### Confocal microscopy, image processing and analysis

Confocal images on fixed samples were acquired using a Nikon A1plus microscope. Z-stacks of cells were acquired from 1 μm below the basal cell surface to 1 μm above the apical cell surface, with a step size of 0.5 μm at 60x magnification. The laser power, pinhole size and other standard parameters were kept identical for all samples. ImageJ software was used for quantification of signal intensities. For quantifying the junctional cadherin intensity, a maximum intensity z projection was performed selecting all the slices in the stack and tracing the cell-cell junctions with the freehand line tool, with a line width of 3 pixels. Using the plot profile function, the intensity plot was obtained and the intensity values were listed and saved in Excel sheets for data processing. In a similar manner, data was collected from the cell free area of the coverslip and subtracted to eliminate the background.

### Tissue culture, cell dissociation

Cells were maintained in high glucose DMEM with 10% FCS, and confluent cultures were routinely treated with TE buffer (0.05% trypsin, 0.02% EDTA). For force measurements, cells were treated with TE buffer and were seeded as single cells in 60-mm ultra-low adhesion dishes (Corning) at 30,000 cells in 3 ml culture medium. After 18 hrs of suspension culture, cells were gently re-suspended in CO_2_-independent medium (Invitrogen) supplemented with 10% FCS and used immediately for SF measurement experiments.

### Measurement of separation force by pillar-pipette assay

A 60-mm ultra-low adhesion dish (Corning) was prepared by cutting a 2-cm window on the side wall for pipette access. A PDMS block (Sylgard, Dow-Corning) with pillars 35 μm in diameter and 200 μm long arranged in a hexagonal pattern with a pitch of 400 μm was cut into a thin slice of 1–2 mm thickness and 1-cm long. The strip was further cut into 5 pieces of ~2-mm thickness and stuck to the pre-prepared ultra-low adhesion dish in a staggered arrangement, so that ~20 micropillars at different x, y, z-levels were available to enable high-throughput. The dish with micropillars was plasma treated to make the pillars hydrophilic (Harrick Plasma). During plasma treatment, a thin film of PDMS was used to mask the area of the dish surface where the ultra-low adhesion property was to be maintained in order to prevent cells from adhering during the experiments; thus selectively plasma treating the PDMS block. After plasma treatment, the thin film of PDMS was peeled off. The PDMS pillars were then coated with 50 μl fibronectin (Sigma-Aldrich) mixed with fluorescently labeled fibronectin (Cytoskeleton) in a ratio of 10:1 and incubated for 1 hr at 37°C. The fibronectin was then washed with deionized water three times and the surface left to dry. The thin film of PDMS was removed from the dish surface before experiments. Cells were manipulated at 37°C with a micropipette that was held by a micromanipulator connected to a combined hydraulic/pneumatic system. Micropipettes were pulled (P-2000, Sutter Instrument) and cut with a microforge instrument (MF-900, Narishige) to an inner diameter of 4–6 μm. Cell doublets were visually observed under bright-field and epifluorescence to identify doublets with mature, bright junctions and a larger contact radius. Such doublets were gently picked up by one end of the doublet using the micropipette. The other end of the doublet was attached to a PDMS pillar tip with the doublet axis parallel to the dish and perpendicular to the pillar’s axis. As soon as the cell starts to adhere and attach to the pillar (2 min), the micropipette was disengaged from the doublet. After 5 min of cell-pillar attachment, a second pre-existing doublet was selected with the micropipette and brought in contact with the first doublet in a series arrangement. The new junction between the two doublets was established over 1 hr. The two doublets were separated from each other using the micropipette while the deflection in the pillar was imaged in bright-field (40× x 1.5, Nikon, Eclipse 2,000) at the rate of 1 image per sec. The SF required to separate the two doublets (after 1 hr contact) was quantified from the pillar deflection using the following equation: SF = (3*EπD*43δ)/(64*L*^3^) (1), where *L* is the effective length of the pillar, *D* is the diameter, *E* is the Young’s Modulus of the PDMS material, and δ is the maximum pillar deflection before detaching the two doublets from each other. Upon detaching the doublets, the two cells of the other doublet still attached to the pillar were separated using the micropipette, and the force required to separate the cells (pre-existing doublets) was determined. The results from 15 measurements from each group were used to obtain the mean SF for a specific contact time in at least three independent experiments.

### Dispase-based junctional protein tensor assay (D-JPT)

Channels made of UV curable polymer (NOA 73, Norland Products) were prepared using a PDMS stencil. A PDMS stencil with the shape of the channel projecting out (100–500 μm high) was placed upside-down on a glass-bottomed dish (IWAKI), and the low viscous UV curable polymer was made to flow through the channels by capillarity effect and then UV cured. The PDMS stencil was peeled off to expose the channel. The channel was washed three times by a jet of deionized water. Cells were seeded at 60% confluence in the rectangular glass-bottomed channel, 1-mm wide and 2.5-cm long with a central gradual constriction of 0.5 mm and 2.5 mm long. Cells were seeded within 3 hrs of making the channels to retain their hydrophobic characteristics to avoid cells attaching to the polymer. Upon reaching >95% confluence, cells that had attached to the polymer, if any, were scrapped off with the back of a 10-μl pipette tip without damaging the cells inside the channel. Thereafter, a circular PDMS block with a central 0.5 cm × 0.5 cm window was inserted onto the dish tightly and the medium from within the window was pipetted out gently as to not damage the cell sheet in the channel. A 250-μl dispase solution (2.4 U/ml) was then gently pipetted into the opening and imaging at 20× was started in an inverted microscope (DMi8, Leica) acquiring 1 image per minute for 45 min to 1 hr. Upon dispase treatment, the cells in the window region lifted off the substrate while constrained at the ends of the long-axis. As the edge of the cell sheet is not constrained in the short-axis of the channel, the sheet thinned out in the middle region due to the cells’ inherent cortical tension. The constriction in the middle of the channel makes the cells in that region more vulnerable to tension, causing the cells in this area to stretch at the expense of contraction of the cells on each side of the constriction.

### Cortical tension measurement

Cells were dissociated from confluent cultures by washing once with complete medium to remove loosely attached cells, and then gently flushing a spot of the monolayer to remove a patch of cells. This was done using a 1-ml pipette containing 1 ml CO_2_-independent medium supplemented with 10% FCS and flushing the spot three times with the same 1 ml of medium. The area from which cells were removed was observed under the microscope to ensure that there were no more cells in the area and thus the isolated cells did not represent a subpopulation of easily detachable cells with different rheology. The dissociated cells were gently pipetted 5 times to dissociate cell clumps before being deposited on a PLL-PEG-coated (SuSoS), glass-bottomed dish (IWAKI) and incubated at 37°C. A 1-cm window for pipette access was cut on the side of the glass-bottomed dish before cell seeding. Micropipettes were pulled as described earlier, and the pulled tip was cut to result in inner tip diameter ranging from 6 to 9 μm. Before each measurement, the pressure in the pipette was equilibrated with the pressure in the dish by monitoring the movement of cells when the pipette was brought closer to the cells. Cortical tension measurements were commenced 30 mins after the deposition of the cells onto the dish; this time delay allowed cells to become spherical at 37°C. Single cells were gently aspirated into the pipette with a low pressure sufficient to grab the cell. The pressure was then gradually decreased until a threshold pressure was reached, at which point the cell formed a hemispherical protrusion into the pipette equal to the radius of the pipette. For measuring the cortical tension of doublets, the tip of the doublets was aspirated. The surface tension was computed using the Young-Laplace law *γ* = P_c_ / (2/R_p_− 2/R_c_), where P_c_ is the negative pressure inside the pipette, R_c_ is the radius of the cell and R_p_ the radius of the pipette [30]. Experiments were performed within an environmental chamber maintained at 37°C.

### G-LISA assay

To quantify active Rac1 and Cdc42 levels, cells were seeded at 50% confluence in a 10 cm cell culture dish. Upon reaching >95% confluence, scratch wounds were created using a 10-μl pipette tip, creating horizontal and vertical wounds at a spacing of ~1 cm. The cultures were washed 3 times with 1x PBS (with calcium and magnesium) to remove the detached cells and incubated for a further 3 h to allow cells to start migrating, a process which is known to be regulated by Rho GTPases. The cells were then lysed, and G-LISA assay was performed as per the manufacturer’s instructions (Rac1 and Cdc42 G-LISA activation assay kits, Cytoskeleton).

### Preparation of wound-free gaps for cell migration assay and analysis

Wound gap (500 μm) masking inserts (Ibidi) were placed in the middle of the wells of 12-well cell culture-treated plates. Cells were seeded by pipetting 70 μl of cell suspension at 6 × 10^5^ cells/ml. After 18–24 h of culture, when the cells had reached >95% confluence, the inserts were carefully removed without disturbing the cells. The wells were gently washed three times with 1 ml PBS (with calcium and magnesium) to remove loose cells. Fresh medium (1 ml) was added to the culture, and the gap was imaged at different locations to acquire an image every 20 min using an inverted microscope (DMi8, Leica) equipped with an environmental chamber maintained at 37°C and 5% CO_2_.

### Statistical analysis

All statistical analyses were performed with GraphPad Prism 5 software using an unpaired, two-tailed Student’s *t*-test. Data are presented as mean ± SD.

## Results

### Cadherin/catenin complex recruitment and epithelial integrity is largely independent of EEC1-3 (but not of EEC1-4) domains

**Fig 1:**
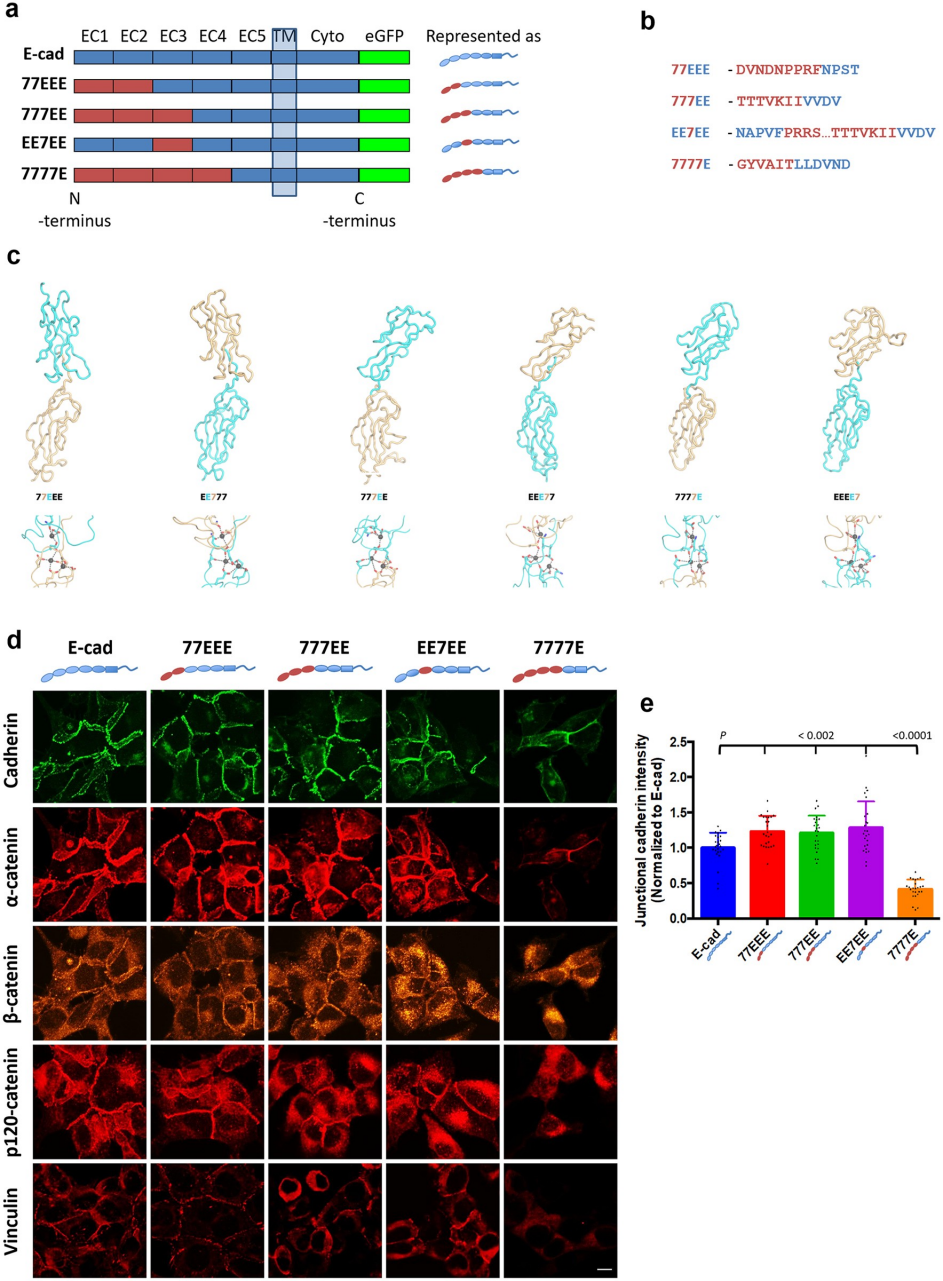
Wild-type and chimeric cadherins used in this study and their colocalization with catenin complexes. **(a)** Schematic representation of the wild-type E-cadherin (E-cad) and chimeras with the EEC1-EEC2, EEC1-EEC3, EEC3 and EEC1-EEC4 swapped with the respective 7EC domains. The EC5 domain, transmembrane domain (TM) and cytoplasmic (Cyto) domain of all chimeras are of E-cad. Wild-type E-cad and chimeras are tagged with eGFP at the C-terminus. The cartoon on the right shows the arrangement of E-cad and cad-7 domains in the wild-type and chimeric cadherins; this will be used in all subsequent figures for easy reference. **(b)** Amino acid sequence at the E-cad–cad-7 chimeric interface. Note- calcium binding sites are preserved. **(c)** Computer models of juxtaposed domains at the hybrid interfaces. Upper panel shows models of the hybrid interfaces between cadherin-7 (7, gold) and E-cadherin (E, cyan). Lower panel shows residues coordinating the predicted calcium ions. Main chain interactions with the calcium ions are not included for clarity. **(d)** Immunofluorescence microscopy of S180 cells expressing eGFP-tagged wild-type E-cad, 77EEE, 777EE, EE7EE and 7777E chimeras, stained with mouse anti-α-catenin (α-catenin), anti-β-catenin (β-catenin), anti-p120-catenin (p120-catenin) and anti-vinculin (Vinculin) antibodies (presented in their original intensities). Note the colocalization of cadherins, α-, β-, and p120-catenins and vinculin at the cell-cell junctions in E-cad, 77EEE, 777EE and EE7EE expressing cells. In the 7777E-expressing cells, cadherin and α-catenin intensity are low at junctions when compared with other cells. β- and p120-catenins in 7777E-expressing cells are more diffusely distributed along the junctions. Vinculin colocalization is not observed in 7777E-expressing cells. Cytoplasmic vinculin is more abundant in 777EE- and EE7EE-expressing cells than in E-cad- and 77EEE-expressing cells. Scale bar is 10 μm. Note: images of cadherin, α-catenin and β-catenin are acquired from same samples. **(e)** Histogram of junctional cadherin intensity normalized to junctional E-cad intensity. Note that the intensity of 77EEE, 777EE and EE7EE are significantly higher than that of E-cad; the intensity of 7777E is significantly lower than E-cad (*n* = 25 in each group, *n* is the number of junctions analyzed).

To study the activities of the EEC domains, we designed chimeric mouse E-cad–cad-7 ectodomains—(N)77EEE(C), 777EE, EE7EE and 7777E—connected to the transmembrane and cytoplasmic regions of E-cad tagged with eGFP at the C-terminus (Fig 1a and 1b). Computer models of juxtaposed domains at the hybrid interfaces showed similar domain orientations between chimeras at each interface (e.g. compare 77EEE versus EE777) suggesting that the design of the chimeras did not disrupt domain:domain interactions (Fig 1c, top panel). Furthermore, analysis of the calcium-binding site demonstrated that at least three sidechains were available to coordinate each calcium ion, supporting that the domain interfaces are stabilized by calcium in a similar manner to the native proteins (Fig 1c, bottom panel) [34]. These chimeras and wild-type E-cad (EEEEE) were stably expressed individually in S180 cells using a lentivirus-based transduction technique, selected by Puromycin resistance and sorted using fluorescence activated cell-sorting (FACS) to pool eGFP positive cells (S1 Fig). S180 untransfected cells were used as a control.

We examined the colocalization of junctional cadherins with catenins and vinculin by immunofluorescence labelling (Fig 1d). We observed that α-, β- and p120-catenins were expressed in all clones and co-localized with cadherins in E-cad, 77EEE-, 777EE- and EE7EE-expressing clones. In 7777E-expressing cells, however, catenins were more diffusely distributed (Fig 1d). Vinculin expression showed a similar trend (Fig 1d).

We determined cadherin-eGFP intensity at cell-cell junctions in 2D culture by confocal microscopy on fixed samples, and found that junctional cadherin-eGFP intensities in 77EEE, 777EE and EE7EE clones were significantly higher than that of E-cad clones (*P* < 0.002; Fig 1e). However, junctional levels of 7777E was significantly lower than that of all the other groups (*P* < 0.0001; Fig 1e). These results show that the cadherin, catenin, and vinculin junctional recruitment hardly depends on EEC1-3. This suggests that the specifics of strand-swapping of type-I or -II cadherins may not be involved.

To verify the importance of strand-swapping, we stably expressed strand-swap incompetent, full-length E-cad mutants (W2A). These cells did not form stable junctions in 2D cultures (S1 Movie, left panel), like the 7777E cells. Thus, epithelial integrity required strand-swapping *per se* independently of the cadherin-type. We thus sought to determine the specific roles of E-cad EC1-3.

### EEC3 accounts for half the strength of mature cell-cell separation force and synergizes with EEC1,2, for fast junction maturation

**Fig 2:**
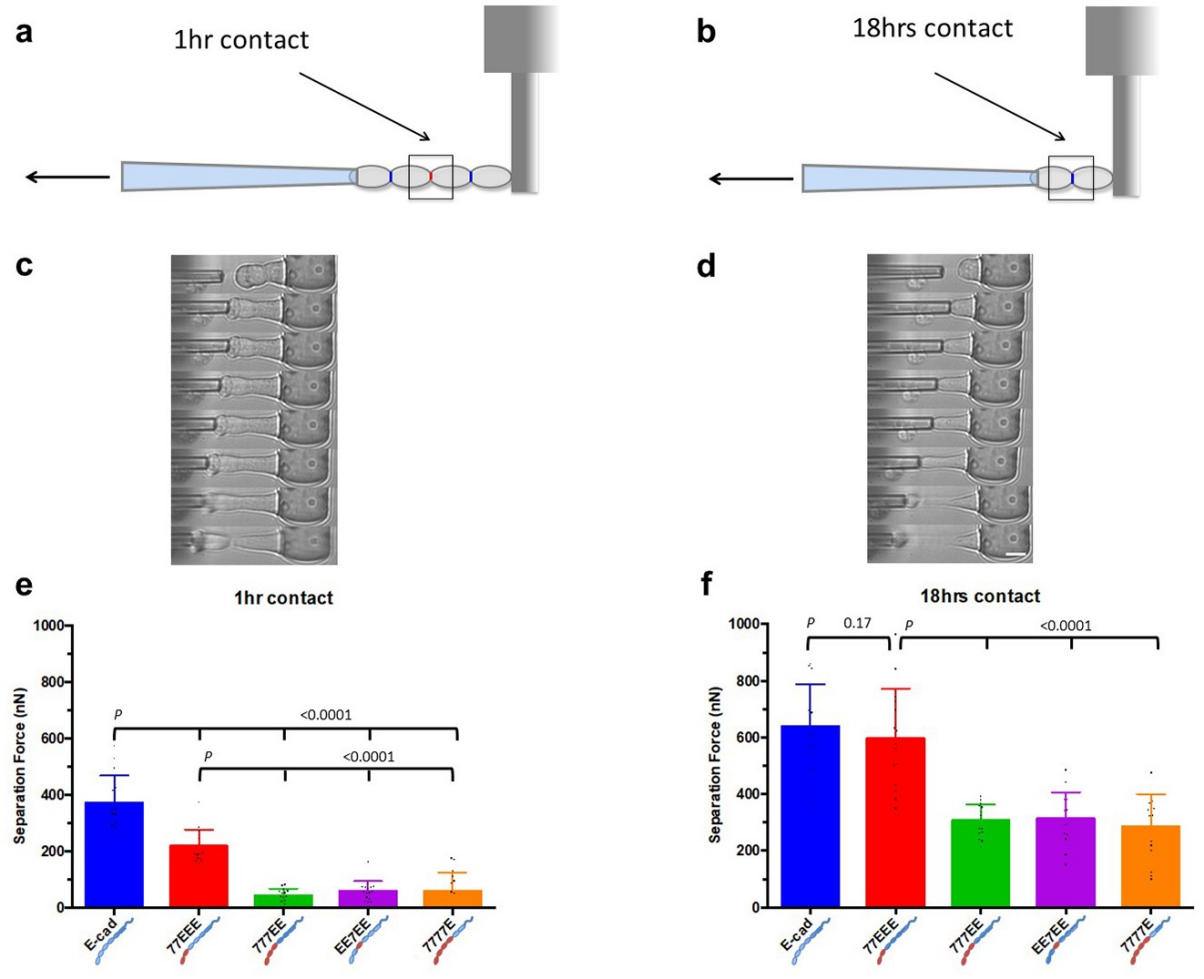
Pillar-pipette assay. **(a)** Cartoon showing two pairs of pre-existing (18 hrs) doublets adhering to each other through a junction established after 1 hr (red, shown by the square box). The doublets are attached to a pillar tip and are pulled by a micropipette to the left. **(b)** Once the two pre-existing doublets are separated from each other, the two cells of the pre-existing doublet with the intact junction (blue, shown by the box) still attached to the pillar are separated. **(c)** Representative kymograph from E-cad group showing two pre-existing doublets adhering to each other through a 1-h contact, and the 4-cell complex attached to a pillar tip. Note the deflection in pillars as the cells are pulled by the pipette. **(d)** Representative kymograph from E-cad group showing a pre-existing doublet with a mature contact and attached to a pillar tip. Scale bar, 15 μm. **(e)** Histogram showing the separation force (SF) for cells expressing wild-type E-cad and chimeric cadherins at 1 hr cell-cell contact (*n* = 15 each). **(f)** Histogram showing the SF for pre-existing (18 hrs) doublets expressing wild-type E-cad and chimeric cadherins (E-cad, *n* = 15; 77EEE, *n* = 15; 777EE, *n* = 14; EE7EE, *n* = 14; 7777E, *n* = 15).

To quantify the SF of cell-cell junctions, we used a novel pillar-pipette assay, where one of the pipettes of the classical dual-pipette assay is replaced by fibronectin-coated polydimethylsiloxane (PDMS) pillars placed perpendicular to the pipette axis and parallel to the imaging plane of an inverted microscope (Fig 2a and S2a–S2c Fig). Mature, pre-existing doublets formed over 18 hrs in suspension culture (pre-existing doublets) in ultra-low adhesion dishes at a low cell density were selected and attached to the side of a pillar tip, and a second doublet was made to adhere to the first doublet in series (Fig 2a and 2b and S2d Fig, top panel). The cell-cell junction between the two doublets matured over 1 hr; this is an optimal timescale for cells from all groups to form sufficiently strong cell-cell adhesions that cause pillar deflection during cell-cell separation while avoiding cell detachment from the pillar. The two doublets were separated from each other using a pipette by aspirating the free end of the 4-cell complex and manually moving the pipette along the y-axis and away from the pillar. This is performed using a micromanipulator at an average speed of ~3 μm/sec, while acquiring images at a rate of ~1 FPS to record the deflection in the pillar (Fig 2c and S2d Fig, bottom panel). Thereafter, the two cells of the pre-existing doublet still attached to the pillar are similarly separated (Fig 2d and S2 Movie). SF after 1 hr contact and pre-existing (18 hrs) contact were quantified from the respective pillar deflections (Equation-1, see Materials and Methods).

At short times (1hr), the SF for all chimeras missing the EEC3 (777EE, EE7EE, 7777E) was reduced by 8 fold compared to wild-type E-cad (*P <* 0.0001) (Fig 2e and S2 Movie). In contrast, 77EEE junctions displayed a 2- fold reduction only. This suggests that EEC3 is essential for stronger SF after short-term contacts.

At longer times (18h) the SF strengthened for every chimeric cadherins. Compared to wild-type E-cad, only 777EE, EE7EE and 7777E displayed a 2-fold reduction in SF (*P* < 0.0001), while 77EEE was indistinguishable (*P* = 0.17) (Fig 2f and S2 Movie). This suggests that SF matured faster by strand-swapping in EEC1 than in 7EC1, provided that EEC3 is present. In sum, the EEC3 is essential for strong cell-cell SFs, and together with EEC1,2 for fast strengthening.

### EEC1-3 and EEC4 antagonistically modulate single cell cortical tension

In the cell-cell SF assay, we noticed that cells expressing wild-type E-cad and 7777E showed larger deformations during separation as compared with 77EEE, 777EE and EE7EE (S2 Movie). Since cell cortical tension may affect cell deformation during this assay, and that intercellular adhesion strength depends on cell cortical tension [28, 29], we reasoned that different cadherin EC domains may have distinct effects on cortical tension.

**Fig 3:**
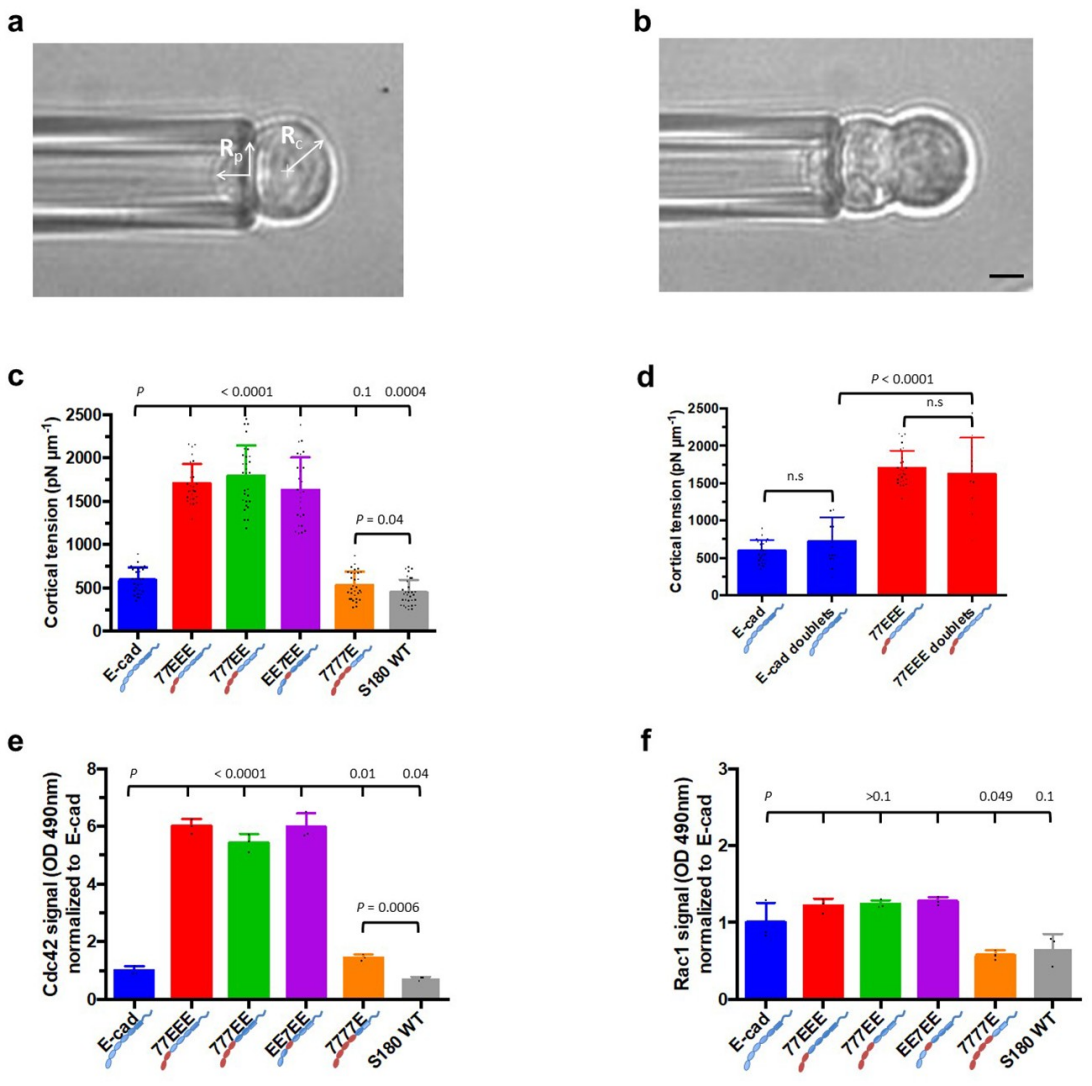
Cell cortical tension and Rho GTPase activity. **(a)** Bright-field image of a single cell held at the pipette by reducing the pressure inside the pipette such that the length of protrusion of the cell inside the pipette is equal to the radius of the pipette R_p_. R_c_ is the radius of the cell. Scale bar, 5 μm. **(b)** Bright-field image of a cell doublet held at the pipette by reducing the pressure inside the pipette such that the length of protrusion of the cell inside the pipette is equal to the radius of the pipette R_p_. Scale bar, 5 μm. **(c)** Histogram of cortical tension values of cells from all groups and the parental S180 wild-type (WT) cells with no endogenous cadherins. Note that cortical tensions of E-cad- and 7777E-expressing cells are significantly higher than that of parental S180 WT cells; cortical tensions of 77EEE-, 777EE- and EE7EE-expressing cells are about three times higher than the parental S180 WT cells. (E-cad, *n* = 28; 77EEE, *n* = 27; 777EE, *n* = 29; EE7EE, *n* = 26; 7777E, *n* = 31; S180 WT, n = 31). **(d)** Histogram comparing cortical tension of single cells and doublets of E-cad- and 77EEE-expressing cells. Following the trend of single cells, the cortical tension of 77EEE-expressing doublets is significantly higher than that of E-cad-expressing doublets. Note that the difference in cortical tension between single cells and doublets is not significant (n.s.) within the same group. However, the spread of values is wider for doublets (E-cad, *n* = 15; 77EEE, *n* = 11). **(e)** Histogram of active Cdc42 signals normalized to E-cad group, showing a 5- to 6-fold higher level of active Cdc42 in 77EEE, 777EE and EE7EE groups. The active Cdc42 in E-cad-expressing cells is significantly higher than that of the parental S180 cells but significantly lower than that of the 7777E-expressing group (*n* = 3). **(f)** Histogram showing active Rac1 levels normalized to the E-cad group. Note that the active Rac1 levels in the 7777E group were marginally lower than those in the E-cad group. The levels in 77EEE, 777EE and EE7EE groups were higher than that in the E-cad group but not statistically significant (*n* = 3).

To test this, we used the micropipette aspiration technique (Fig 3a and 3b) to characterize the cortical tension of single cells and doublets derived from confluent 2D cultures by trypsin-less dissociation. The cortical tensions of cells expressing chimeric cadherins 77EEE, 777EE and EE7EE were significantly higher than that of cells expressing the wild-type E-cad or the chimeric 7777E (Fig 3c, *P* < 0.0001). The cortical tension of parental S180 cells (devoid of E-cad expression) was significantly lower than that of E-cad–expressing cells. Finally, the difference in cortical tension between E-cad and 7777E cells was much smaller than between these three groups and other chimera-expressing cells. Strikingly, these results show that changing a single, specific EC domain in an intercellular adhesion protein appears sufficient to induce changes in single cell mechanical property.

To assess whether cortical tension also depends on intercellular adhesion, we then measured differences in cortical tension in doublets of E-cad- or 77EEE-expressing cells. We observed no significant difference between cortical tension of single cells and doublets of the same group (Fig 3d, *P =* 0.07 and *P =* 0.5 respectively), and the difference in cortical tension between E-cad and 77EEE doublets was significant (Fig 3d, *P* < 0.0001). Therefore, cortical tension depends on the EC regions of expressed cadherins but not on intercellular adhesion.

### Cortical tension follows GTPase signaling

To investigate how cadherin EC region may affect cell cortical tension, we examined the activities of Cdc42 and Rac1 using a G-LISA assay on cell lysates. Indeed, distinct Rac1 activity—both cell-autonomous and cadherin *trans*-bond-dependent—occurs in response to the expression of different cadherin proteins in the same cellular background [38]. When compared with E-cad–expressing cells, we found a substantial increases in the active Cdc42 levels in cells expressing 77EEE (*P* < 0.0001; Fig 3e), 777EE (*P* < 0.0001), EE7EE (*P* < 0.0001). Cells expressing 7777E also exhibited an increase (*P* = 0.01), albeit much smaller. Active Cdc42 levels were also low in the parental S180 cells (*P* < 0.04). Active Rac1 levels of all the cell lines, however, were not significantly different from that of the E-cad expressing cells (*P* ≥ 0.1), with the exception of 7777E-expressing cells, which was lower (*P* = 0.049) (Fig 3f). These results show that Cdc42 activity levels correlate with cell cortical tension.

### Cell-cell separation forces are governed by dissipative processes

**Fig 4:**
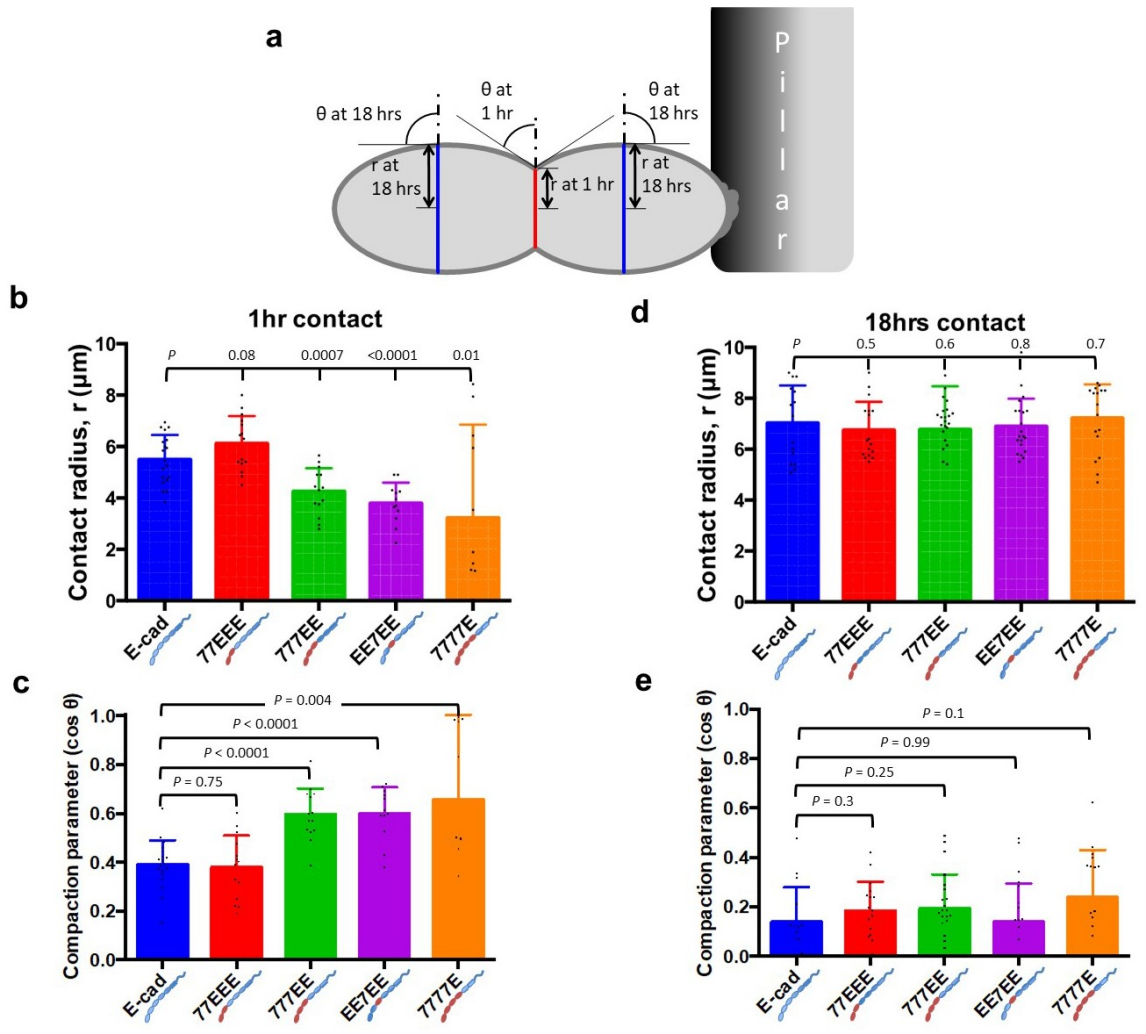
Contact radius and compaction parameter. **(a)** Drawing depicting two pre-existing (18 hrs) doublets adhering to each other through a 1-h contact. The 1-h cell-cell junction is shown in red and the pre-existing (18-h) junctions in blue. Measurements of the cell-cell contact angle “θ” and the contact radius “r” in 1 hr and pre-existing (18 hrs) doublets are illustrated. **(b)** Histogram of contact radius at 1 hr contact (E-cad, *n* = 19; 77EEE, *n* = 15; 777EE, *n* = 14; EE7EE, *n* = 12; 7777E, *n* = 10). **(c)** Histogram of dimensionless compaction parameter (cos θ) at 1 hr of contact (E-cad, *n* = 19; 77EEE, *n* = 14; 777EE, *n* = 14; EE7EE, *n* = 12; 7777E, *n* = 10). Note that, although the compaction parameter of E-cad and 77EEE groups are similar, their SFs are significantly different, as seen in Fig 2e and 2g. **(d)** Histogram of contact radius in pre-existing (18 hrs) doublets (E-cad, *n* = 15; 77EEE, *n* = 17; 777EE, *n* = 23; EE7EE, *n* = 21; 7777E, *n* = 17). Note that the mean contact radii of all groups are similar, although their separation forces (SFs) are significantly different (Fig 2f and 2h). **(e)** Histogram of compaction parameters of pre-existing (18 hrs) doublets (E-cad, *n* = 15; 77EEE, *n* = 17; 777EE, *n* = 23; EE7EE, *n* = 21; 7777E, *n* = 17). Note that the compaction parameters for E-cad and other groups are similar, even though they show significantly different SFs (Fig 2f and 2h).

Within a previously established theoretical framework [8], it is possible to assess the contribution of static intercellular adhesion energy to cell-cell SF. Indeed, the static intercellular adhesion energy W is defined as W = γ(1-cosθ), where γ is the cortical tension and cosθ the compaction parameter (Fig 4a). In turn, the static SF, SF_static_ = πRW, where R is the cell radius, in the limit of low compaction. Finally, the contact radius r is determined by r = Rsinθ. Thus, we set out to measure cell-cell compaction and the contact radius.

At 1 hr of contact, the trends of the mean contact radius and compaction parameter were seemingly indicative of the measured SF across different groups (Fig 4b and 4c). Indeed, E-cad- and 77EEE-expressing cells showed insignificant differences in contact radii and compaction parameters that were higher and lower, respectively, than that of the other chimeric EC cells. The SF, r and cosθ metrics appeared to behave consistently with higher intercellular adhesion energy in E-cad- and 77EEE-cells compared with other chimeric EC cells. After 18 hrs, however, both the contact radius and compaction parameter narrowed to around 7 μm and 0.18, respectively, for all groups, without significant differences between groups (Fig 4d and 4e).

We next computed the SF_static_ from cortical tension and compaction parameters at 1 hr (where low compaction is a reasonable assumption) and 18 hrs. We found that SF_static_ scaled well below the measured SFs in all conditions and, notably, at least an order of magnitude below that measured in cells expressing E-cad or 77EEE (Table 1). We conclude that static adhesion energy only marginally contributes to cell-cell SFs and that EEC1-3 contributions to cell-cell SFs essentially arise from dissipative processes.

**Table 1:** Comparison between measured separation force and computed static separation force (SF vs SF_Static_).

	Measured								Calculated					
	SF (nN)		R (μm)		Cosθ		γ (pN /μm)		R = r/sqrt(1- Cos^2^θ		W = γ (1-Cosθ)		SFs = πRW	
Hour	1	18	1	18	1	18	1	18	1	18	1	18	1	18
E-cad	373	639	5.5	7	0.4	0.15	590	722	6.001	7.080	354	613.7	6.674	13.650
77EEE	218	598	6	7	0.4	0.18	1,703	1,627	6.546	7.116	1,021.8	1,334.14	21.015	29.826
777EE	44	307	4.5	7	0.6	0.18	1,792		5.625	7.116	716.8		12.667	
EE7EE	59	314	4	7	0.6	0.13	1,629		5	7.059	651.6		10.235	
7777E	59	286	3	7	0.62	0.21	529		3.823	7.159	201.02		2.415	
S180							446							

### EEC3 and EEC1-3 provide tensile strength and ductility, respectively, to epithelial cell sheets

**Fig 5:**
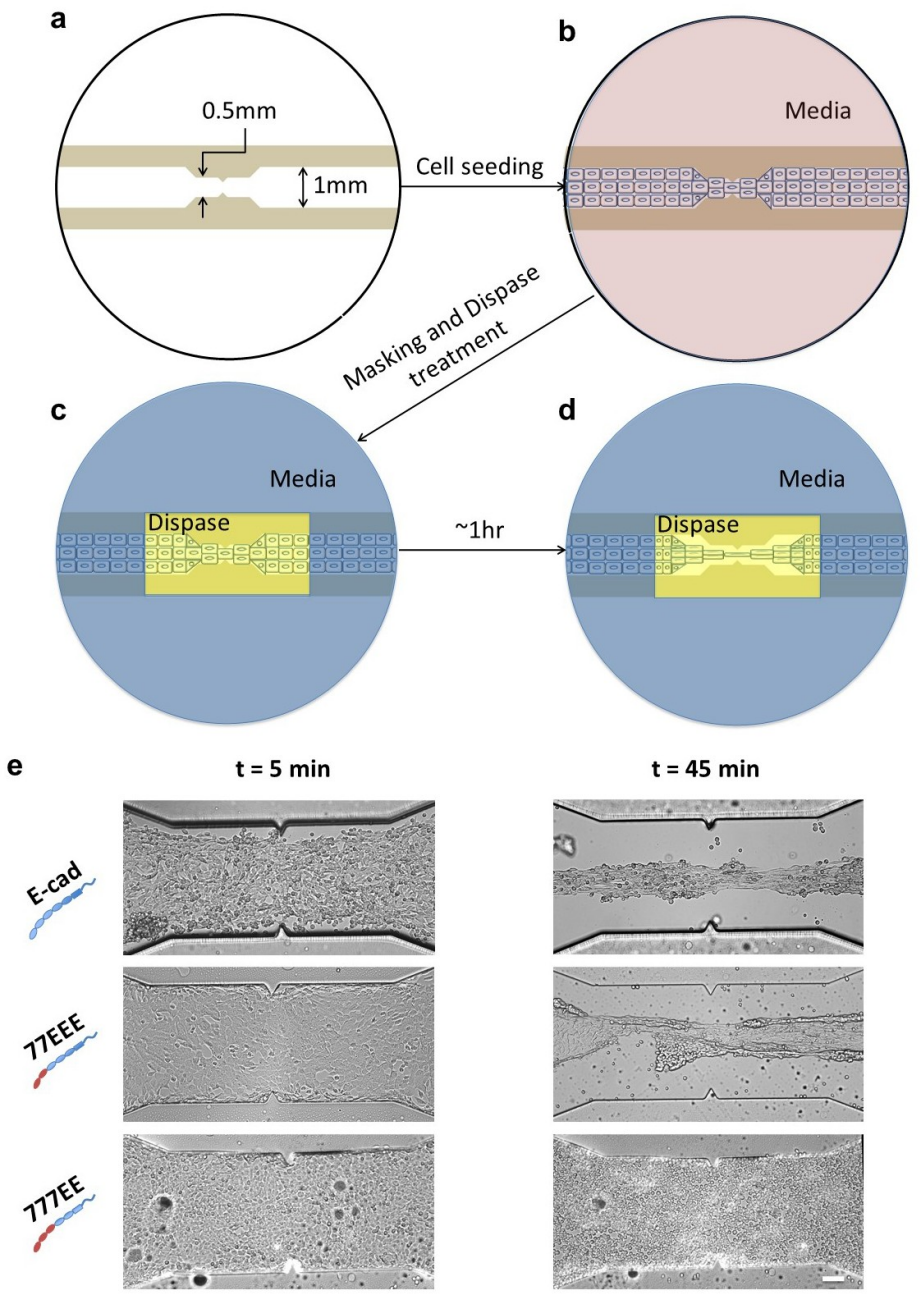
Dispase-based junctional protein tensor assay. **(a)** Schematic of a 1-mm wide channel with a central constriction of 0.5 mm made of UV curable polymer on a 27-mm glass-bottomed dish. **(b)** Channel seeded with cells forming cell-cell junctions. Fewer cells are shown here for ease of representation. **(c)** PDMS mask (blue) with a 5 mm × 2.5 mm opening in the middle that snuggly fits inside the dish. The media is removed from the opening and dispase (yellow) is added. The media remains in the area covered by the PDMS mask. **(d)** After ~1 hr of dispase treatment, cell-substrate bonds within the area are cleaved while the cells under the PDMS, where the culture medium is present, are still attached to the substrate. In the absence of cell-substrate adhesion, the cells that become lifted in the constricted dispase-treated region experience tension and stretch in the direction along the channel at the expense of cells contracting at either side of the constriction due to the intrinsic cortical tension of the cells. Figure not to scale. **(e)** E-cad-, 77EEE-, and 777EE-expressing S180 cells subjected to dispase treatment. Imaging was commenced 5 min after the addition of dispase for 1 hr. Note that, in the t = 45 min image, the E-cad-expressing cells have undergone significant strain yet the cell-cell contacts are still maintained. In the 77EEE-expressing cells, the cell sheet snaps when the sheet is lifted at lower strain levels as compared to E-cad-expressing cells. Although cell-cell junctions are formed similarly in 777EE- and 77EEE-expressing cells, 777EE-expressing cells are not able to maintain cell-cell contact in the absence of cell-substrate adhesion; they became single cells and cell clumps that are unable to withstand the tension as a cell sheet. Scale bar, 100 μm.

Given the uncorrelated effects of the EC region on cell-cell SFs and cortical tension, we sought to assess how this combination would affect the mechanical properties of epithelial tissues. To avoid the confounding effects of cell-substrate adhesion, we developed a dispase-based epithelial tissue uniaxial stretcher that locally cleaves cell-substrate bonds, causing a local stretch on the adherent cell sheets in culture, and thereby shifting cell-generated tension entirely to cell-cell junctions (Fig 5a–5d).

We found that, following dispase treatment, the E-cad cell sheet could withstand cell-generated tension while thinning as a ductile material (Fig 5e, top panel and S3 Movie, top panel). This was accompanied by cell reorientation along the direction of tension. Comparatively, the initially cohesive 77EEE cell sheet soon snapped under dispase treatment—even though the two parts of the sheet each remained cohesive upon tension relief (Fig 5e, middle panel and S3 Movie, middle panel)—whereas the 777EE and EE7EE cell sheets disintegrated to single cells and cell clumps with tethers in between, respectively (Fig 5e, bottom panel, S3 Movie, bottom panel & S4 Movie). Finally, cells expressing 7777E failed to form cohesive cell sheets (S5 Movie), consistently with the inability of this chimera to support epithelial integrity (see above). Considering ductility as the inverse of the sheet cross-section area at rupture, and tensile strength the pulling force at rupture, these results suggest that EEC3 alone provides tensile strength to the cohesive cell sheet, while together with EEC1,2 it provides ductility, so that the sheet can withstand larger strains before rupture.

Since the main phenotypic differences between wild-type and 77EEE cells appeared at the tissue level, in terms of ductility, we sought to determine the relevance of EC1,2-mediated dimerization in this process. As we had previously shown that epithelial integrity was impossible without strand-swapping (S1 Movie), we stably expressed X-dimer incompetent (K14E mutation) full-length E-cad mutants. In contrast with cells expressing strand-swap mutants, which bear no tensile-strength, cells expressing X-dimer-incompetent E-cad formed stable junctions comparable to those formed by cells expressing wild-type E-cad, consistent with an effect restricted to the interaction kinetics. Moreover, the cells showed a ductile behavior like that of wild-type E-cad expressing cells (S6 Movie). Thus, these results show that X-dimerization is neither essential nor accountable for differences in tissue ductility, pointing to a cell-autonomous mechanism, as for cell cortical tension.

### EEC1-3 are essential for collective cell migration speed

Previous studies proposed how cell-cell adhesion and cell cortical tension governed the dynamics of collective behaviours in adherent cell assemblies [39]. Thus, we next sought to assess how altering the material properties of cell sheets with different ECs would impact dynamic multicellular processes using wound-free gap closure assays.

**Fig 6:**
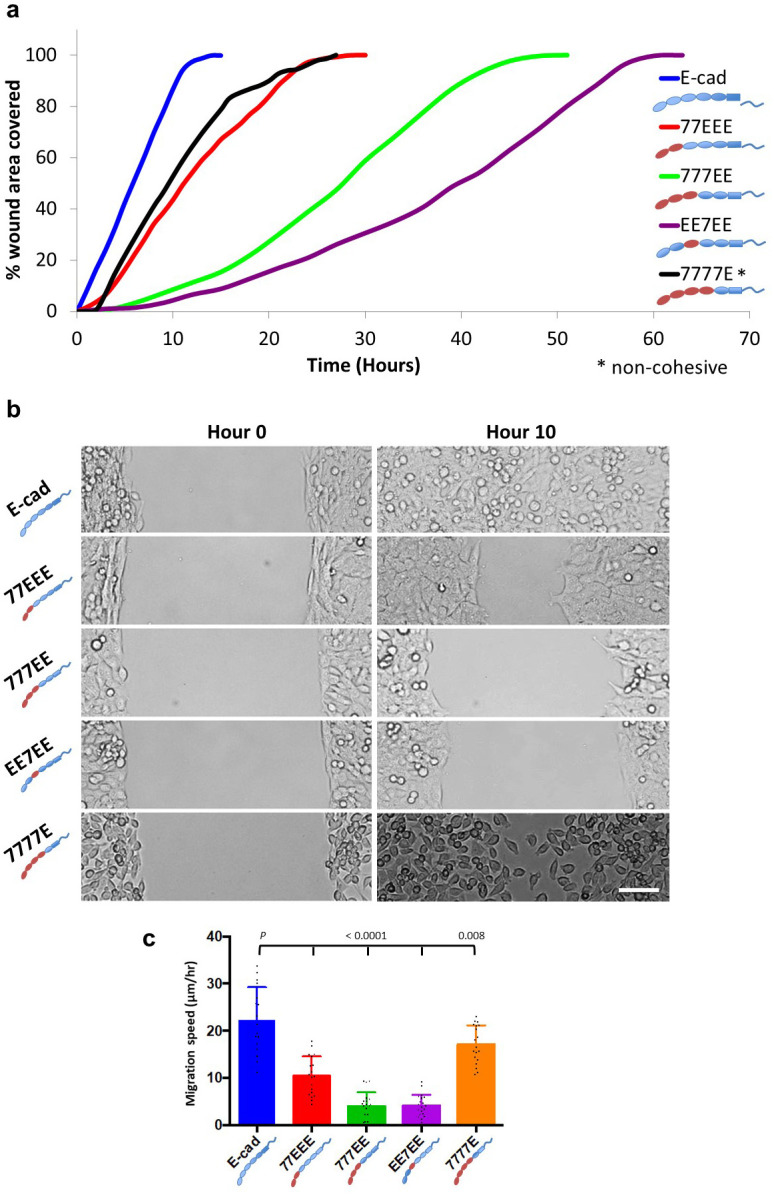
Wound healing assay. **(a)** Representative curves showing the time taken to cover a 500-μm unwounded gap. All groups migrated collectively, except 7777E-expressing cells. E-cad expressing cells migrated fastest, followed by 77EEE-expressing cells. 777EE- and EE7EE-expressing cells took approximately 4- to 5-times longer than the time taken by E-cad-expressing cells. The migration speed of 7777E-expressing cells is comparable to that of E-cad-expressing cells; however, these cells migrated as a non-cohesive cell assembly, as the cell-cell adhesion in these cells is transient. **(b)** Representative images from each group showing the position of cells at the beginning and end of 10 h of migration. Scale bar, 50 μm. **(c)** Histogram showing the cell migration speed of all the groups. (E-cad, *n* = 17; 77EEE, *n* = 19; 777EE, *n* = 20; EE7EE, *n* = 20; 7777E, *n* = 19).

Cells expressing 7777E showed the fastest gap closure compared with the other EC chimera-expressing cells (Fig 6a–6c and S7 Movie) but slower than E-cad expressing cells (*P* = 0.008), and this occurred as a non-cohesive cell assembly. This is consistent with epithelial integrity impeding cell migration speed. Nevertheless, the rate of gap closure for E-cad-expressing cells was twice as fast as that of 77EEE-expressing cells (*P* < 0.0001); while the rate of 777EE- and EE7EE-expressing cells was significantly lower than that of E-cad (*P* < 0.0001) (Fig 6c). Together, these results are consistent with a positive effect of EEC3 on collective cell migration, which provides higher tensile strength, and of EEC1-3, which provides higher ductility, in a manner that compensates epithelial integrity effects caused by EEC4. Remarkably, this verifies previous predictions [39]. A summary of the results is presented in Table 2.

**Table 2:** Summary of results.

	Cadherin expressed	E-cad	77EEE	777EE	EE7EE	7777E
**Phenotype**	**Cell-cell adhesion strength**	***	**	*	*	*
	**Cortical tension**	*	***	***	***	*
	**Collectivity/Colony formation**	***	***	***	***	*
	**Migration speed /Motility**	***	**	*	*	***

The influence of the different chimeric cadherins on the selected phenotypes are classified as *** High, ** Moderate and * Low.

## Discussion

Here, we sought to characterize how the EC domains of type-I and type-II cadherins affect cell-cell adhesion and tissue phenotypes by expressing chimeras of E-cad and cad-7. Unlike previous studies with deletion mutants [22, 24], our approach preserves a full-length protein with inter-EC calcium binding sites (Fig 1c), and the transmembrane and cytoplasmic regions of E-cad. It is thus less prone to potential structural defects. Effects of chimeras did not correlate with their small differences in expression levels (S1 Fig) and are thus attributable to the type of EC displayed.

First, we showed that EC domains regulate the recruitment of cadherins and their cytoplasmic partners in a cadherin-type specific manner that does not rely on the strand-swap mechanism alone but involves EC4 (Fig 1d). Exchanging EC4 of E-cad for that of N-cadherin was previously shown to promote a mesenchymal phenotype [40]. Hypoglycosylation of EEC4 impacts the composition and stability of the cadherin/catenin complex, and impedes collective cell migration [41, 42]. Normal glycosylation of EC4 of VE-cadherin was previously found to prevent cadherin cis-clustering [43]. Whether EC4-based cis-interactions play a role here and whether they are glycosylation-regulated should be the focus of future studies [44]. Unknown interactions involving EEC4 with other membrane proteins may also be at play.

Second, we showed that mature SFs reach similar levels regardless of the type of EC1,2 and are high only in cells expressing cadherins with EEC3 (Fig 2). Our quantitative analysis of cell cortical tension and compaction revealed that the static intercellular adhesion energy only marginally contributes to SFs (Figs 3 and 4, Table 1), which (SFs) are moreover only transiently affected by strand-swap or X-dimerization specifics (Fig 2). Remarkably, EC3 glycosylation of N-cadherin impedes cis-dimerization and *trans*-bond kinetics [45, 46]. Future studies may address whether EEC3, possibly through glycosylation-dependent cis-clustering, contributes to dissipative mechanisms that result in a strong SF. Consistently, we show that EEC3 provides tensile strength to epithelial sheets (Fig 5).

Third, cell-cell separation ultimately involves cadherin-mediated *trans*-bonds rupture. The energy per unit area G associated with the rupture of flexible bonds between two surfaces is reflected by (n_0_/2k)(k_B_T/Δ)^2^ln^2^(v/v_0_), where n_0_ is the bond surface density, k the molecular stiffness of the bond, k_B_ the Boltzmann constant, T the temperature, Δ the interaction spatial range of the bond, v the pulling velocity, and v_0_ the thermal velocity of bond rupture [47]. Using typical values [35] of n_0_~10^16^ m^-2^, k~10^−3^ N/m, Δ~0.1 nm, v~3 μm/s (as in our conditions) and v_0_~0.01 μm/s, G is about 60 mN/m. Scaled to the size R~5 μm of the cell, this gives a contribution to the SF easily reaching several hundred nN, which is in remarkable accordance with our measured values. Cadherin trans-bond rupture is thus sufficient to contribute to dissipation resulting in strong SF, without excluding additional bond ruptures between cadherin complexes and the cytoskeleton, or within the cortex.

Rho GTPases Cdc42 and Rac play a critical role in the maturation of strong SFs [26], as a result of local dissolution of the actomyosin cortex [48, 49]. Moreover, sub-type-dependent cadherin regulation of Rho GTPases may also affect the cortex at a distance, as measured by cell twisting cytometry [38]. We show here in addition that EEC1-3 and EEC4 are involved in antagonistic, cell-autonomous outside-in controls of Rho GTPases and cortical tension (Fig 3). Moreover, our tissue stretching assay reveals a correlation between low cortical tension and tissue ductility, independent of X-dimerization (Fig 5, S6 Movie). This is consistent with the idea that soft cells, cell-autonomously, favor tissue deformation.

Finally, wound-healing assays reveal an apparent complex dependence on cadherin EC domains. Their effects on intercellular contact formation, SFs, cortical tension, tissue tensile strength, and tissue ductility in fact verify theoretical predictions [39] whereby stronger intercellular adhesion (here provided by EEC3) and lower cell cortical tension (here provided by EEC1,2) favor collective cell migration (Fig 6). In this work, we have mostly focused on the cell mechanical effects of EC domains and their consequences on migration. Previously, other studies have evidenced associations between point-mutations in E-cad, EGFR signaling and migration, and we had also shown cadherin type-dependent control of cell mechanics through the Src-PI3K axis [50–53]. Whether EGFR and/or Src-PI3K signaling are involved in the control of cell migration by EC domain-dependent cell mechanics we show here, or through alternate ways may be the focus of future studies, so is whether known human mutations in E-cad may recapitulate EC-dependent cell mechanics and associated altered migration phenotypes [50–53].

## Conclusion

**Fig 7:**
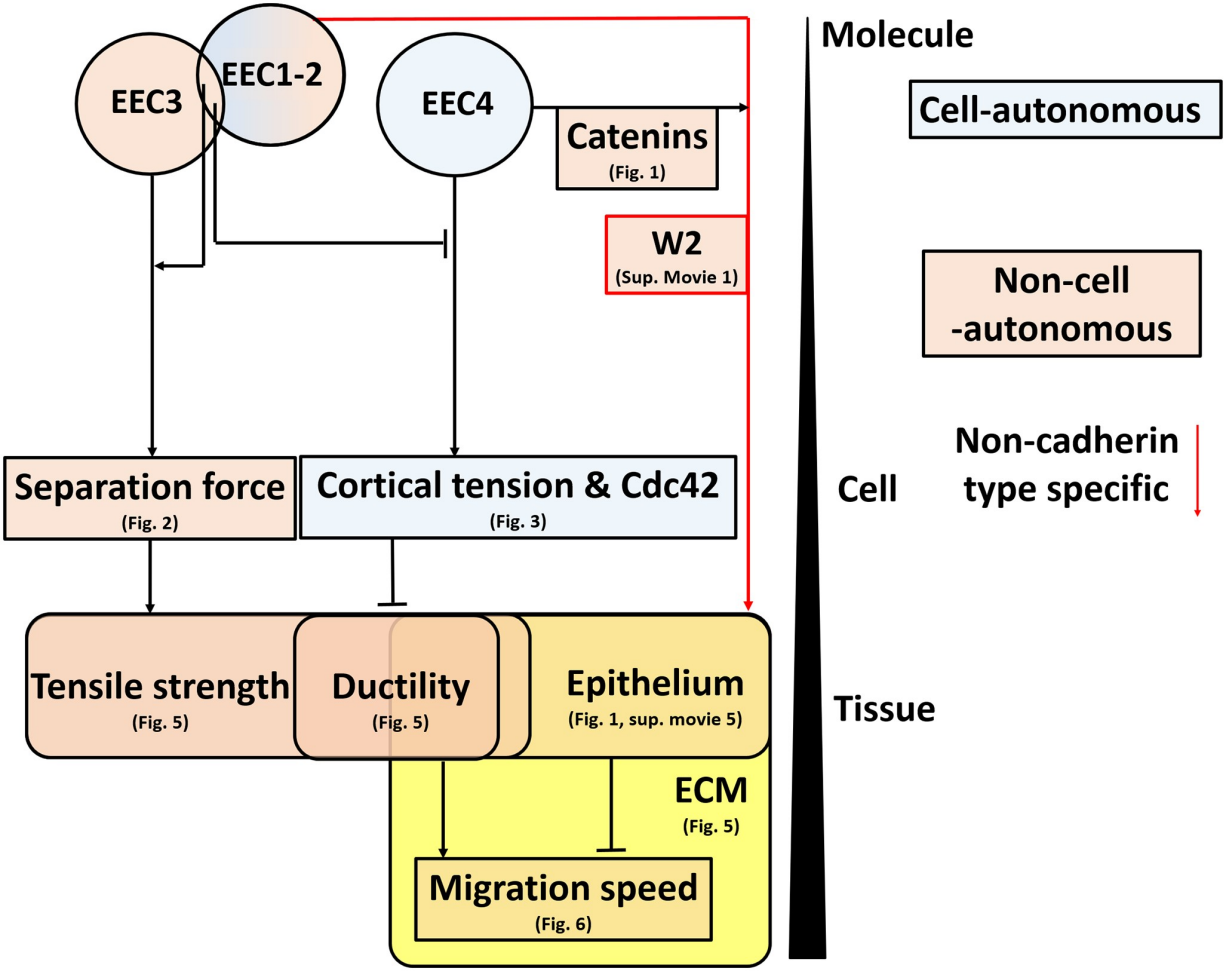
Proposed model depicting the influence of EEC1-4 domains on cell-autonomous and non-cell-autonomous cell and tissue properties. EEC4 regulates catenin recruitment and formation of epithelia on the extracellular matrix, and, antagonistically with EEC1,2 but in a Cdc42-dependent manner, cell–autonomous cortical tension. EEC1,2 also with EEC3 regulate intercellular separation forces together with the cytoskeleton and other cytoplasmic factors. High intercellular separation forces support tissue tensile strength while low cortical tension promotes tissue ductility. Tissue ductility in turn stimulates collective cell migration speed.

In summary (Fig 7), we determined that the EEC4 region promotes the recruitment of catenins and vinculin to cell-cell contacts and epithelium-like cohesive cell sheet formation on an extracellular matrix (Figs 1 and 5, S5 Movie). EEC4 also contributes to the increase in cell cortical tension in an intercellular adhesion-independent manner, likely through a Cdc42 pathway (Fig 3). In turn, the EEC3 is required to provide strong cell-cell SF (Fig 2) and, subsequently, tensile strength of the cell sheet (Fig 5), mostly through an increase in dissipation during cell-cell separation rather than an increase in static intercellular adhesion energy (Fig 4). Moreover, EEC1,2 together with EEC3 provide an additional increase in cell-cell SF and tensile strength (Fig 2, S3 Movie), meanwhile antagonizing EC4 activity on cell cortical tension (Fig 3) for increased ductility to the cell sheet (Fig 5, S3 Movie). Finally, we propose that higher tensile strength and increased ductility compensates the dampening effect of epithelium formation on cell migration speed (Fig 6).

## Supporting information

S1 Fig Flow cytometry data (BD Accuri C6) on cell groups previously FACS sorted based on overall GFP expression. (TIF)

S2 Fig Pillar-pipette assay. **(a)** 3D Computer-Aided Design (CAD) model of 4 PDMS blocks glued to a 60-mm ultra-low dish, cut on both sides to facilitate pipette access. **(b)** Zoomed-in, top view of the dish with PDMS blocks showing pillars (~200 μm long and ~30 μm in diameter arranged in a hexagonal pattern with a pitch of 400 μm) parallel to the x-y plane of the dish and at different z-planes. Note the staggered arrangement of the blocks to facilitate a longer reach of the pipette entering from the left side; the pipette can reach the last pillar on the right extreme without disturbing other pillars along the way. **(c)** A closer look at individual pillars. Note the two pillars at different z-planes. **(d)** Bright-field images showing an undeflected, fibronectin-coated pillar (top), to the tip of which a 4-cell complex is attached and a micropipette accessing the free end of the cell complex. Note that the four cells, two pairs of preformed doublets after 1 hr contact are in series. The pillar deflects as the pipette pulls the cell to the left. The maximum pillar deflection (bottom) is used to calculate the separation force SF using equation (1). Scale bar, 15 μm.
(TIF)

S1 Movie S180 cells expressing strand-swap incompetent (Left) and X-dimer incompetent (Right) E-cad mutants. Note that stable junctions were not formed in the S180 cells expressing the E-cad W2A mutant. Imaged for 6 hrs acquiring 1 image every 20 min. Playback at 4 FPS. Scale bar is 15μm.
(MOV)

S2 Movie Pillar-pipette assay. **(Left)** Representative movies from each group showing two pre-existing doublets adhering to each other at 1 hr contact and the 4-cell complex attached to a pillar tip. Note the deflection in pillars as the cells are pulled by the pipette. The SF values are listed on the left side. **(Right)** Representative movies from each group showing a pre-existing doublet attached to a pillar tip. The SF values are listed on the right side. The pillars are 36.7 μm in diameter and 188.7 μm long (full length not shown). The distance of cell attachment from the tip of the pillar is offset for calculating the effective length. Images were acquired at a rate of ~1 FPS, playback at 4 FPS.
(MOV)

S3 Movie Dispase-based junctional protein tensor assay. **(Top)** E-cad-expressing S180 cells subjected to dispase treatment. Imaging commenced 5 min after the addition of dispase and was imaged for 1 hr, at a rate of 1 image per minute. Note that cells undergo significant strain while the cell-cell contacts are maintained. **(Middle)** 77EEE-expressing S180 cells subjected to similar treatment as in (e). Note that the cell sheet snaps immediately after the sheet is lifted and in lower strain levels as compared with E-cad-expressing cells in (a). **(Bottom)** 777EE-expressing S180 cells imaged for 15 min immediately after the addition of dispase. Although cell-cell junctions and a cell sheet are formed as for 77EEE-expressing cells, the 777EE-expressing cells are not able to maintain cell-cell contact in the absence of cell-substrate adhesion; they become single cells and cell clumps, unable to withstand the tension as a cell sheet. For all three frames, dotted circles show the area that is presented in higher magnification on the right. Playback at 4 FPS. Scale bar, 100 μm.
(MOV)

S4 Movie Dispase-based junctional protein tensor assay of S180 cells expressing EE7EE. The previously cohesive cell sheet disintegrates into cell clumps, forming long membrane tethers in some cells. Note that the cortex of the cells forming tethers is rounded in the middle, indicative of higher cortical tension. Refer to supplementary movie 2 for movie acquisition parameters.
(MOV)

S5 Movie Time-lapse confocal microscopy of S180 cells expressing cadherin-eGFP. **(a)** E-cad-expressing cells **(b)** 77EEE-expressing cells **(c)** 777EE-expressing cells **(d)** EE7EE-expressing cells **(e)** 7777E-expressing cells, imaged for 6 min acquiring 1 image every 20 sec, playback at 4 frames/sec.
(MOV)

S6 Movie Dispase-based junctional protein tensor assay of X-dimer incompetent E-cad K14E mutants. Movie showing randomly oriented S180 cells expressing E-cad K14E mutants aligning along the axis of tension after dispase treatment. Refer to supplementary movie 3 for movie acquisition parameters.
(MOV)

S7 Movie Representative movies from each group showing 10 h of cell migration. Images were acquired every 20 min until the wound-free gap disappeared. Playback at 4 FPS. Scale bar is 100 μm.
(MOV)
