# Supplementary figures and images for: Extracellular domains of E-cadherin determine key mechanical phenotypes of an epithelium through cell- and non-cell-autonomous outside-in signaling
Source: PLoS One. 2021 Dec 22;16(12):e0260593. doi: 10.1371/journal.pone.0260593 (PMC8694416; doi:10.1371/journal.pone.0260593)

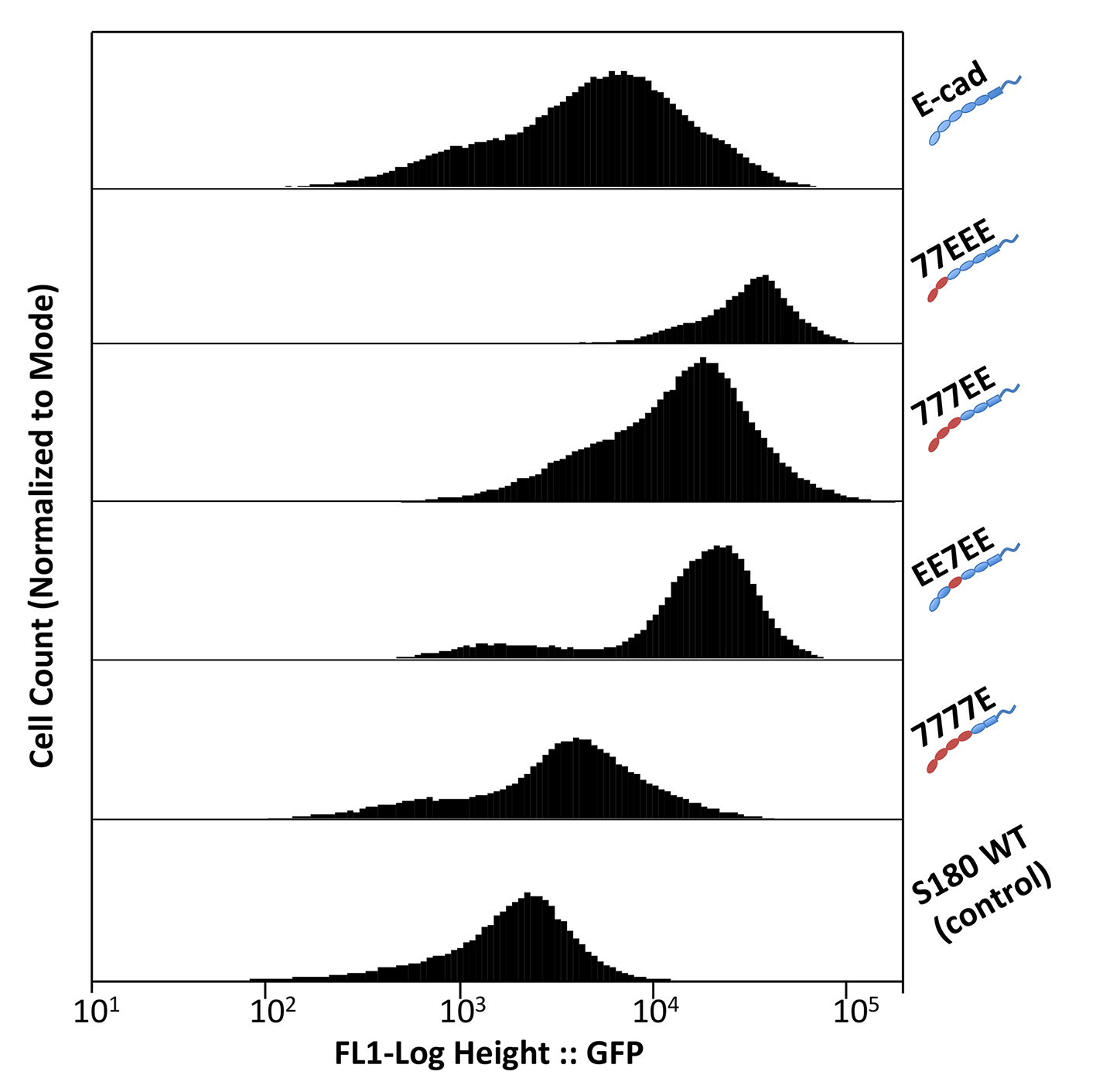

Supplement: S1 Fig — (TIF) [file pone.0260593.s001.tif]

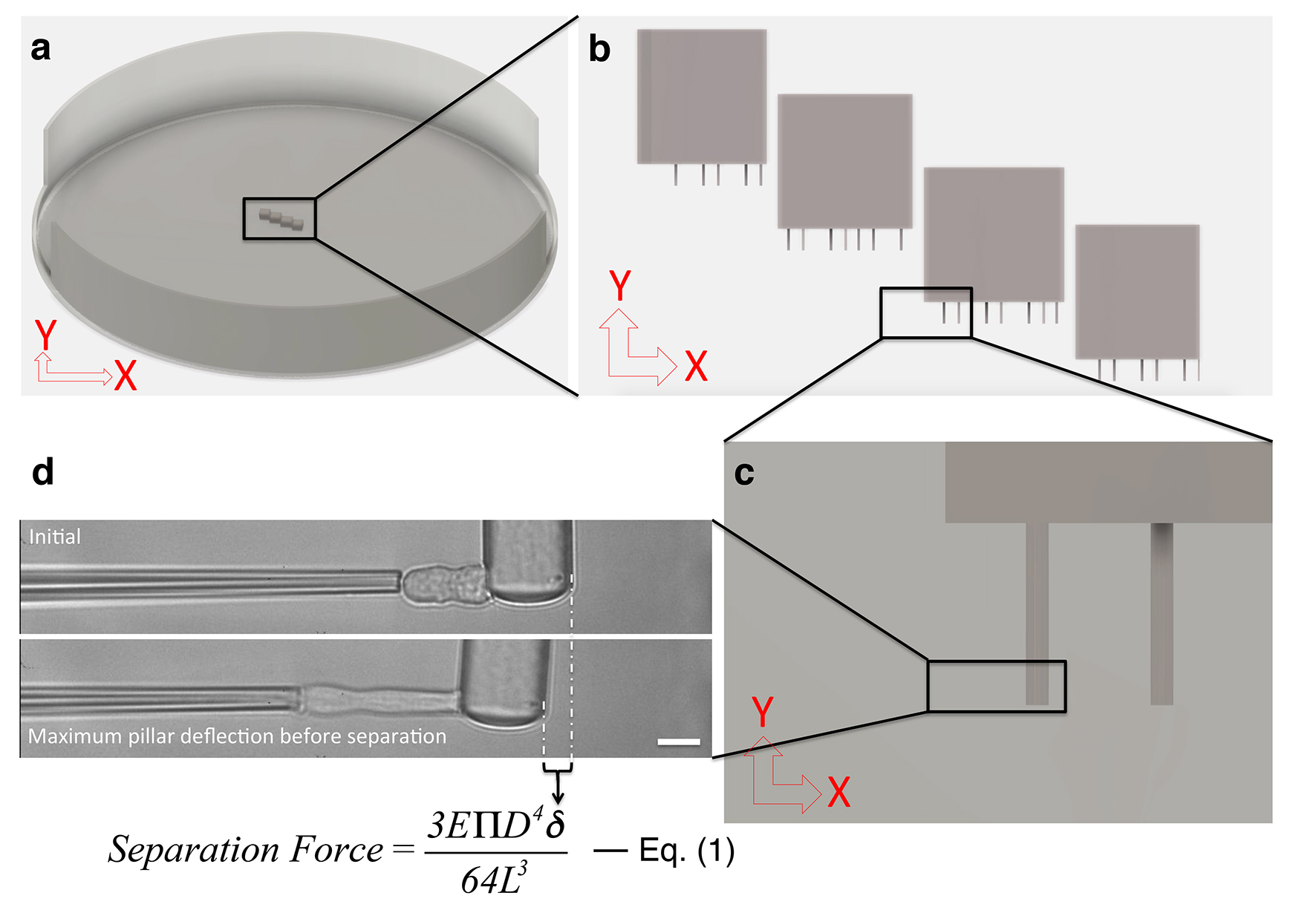

Supplement: S2 Fig — (a) 3D Computer-Aided Design (CAD) model of 4 PDMS blocks glued to a 60-mm ultra-low dish, cut on both sides to facilitate pipette access. (b) Zoomed-in, top view of the dish with PDMS blocks showing pillars (~200 μm long and ~30 μm in diameter arranged in a hexagonal pattern with a pitch of 400 μm) parallel to the x-y plane of the dish and at different z-planes. Note the staggered arrangement of the blocks to facilitate a longer reach of the pipette entering from the left side; the pipette can reach the last pillar on the right extreme without disturbing other pillars along the way. (c) A closer look at individual pillars. Note the two pillars at different z-planes. (d) Bright-field images showing an undeflected, fibronectin-coated pillar (top), to the tip of which a 4-cell complex is attached and a micropipette accessing the free end of the cell complex. Note that the four cells, two pairs of preformed doublets after 1 hr contact are in series. The pillar deflects as the pipette pulls the cell to the left. The maximum pillar deflection (bottom) is used to calculate the separation force SF using equation (1). Scale bar, 15 μm. (TIF) [file pone.0260593.s002.tif]
